# Good Training Should Be Patient-Centred: An Analysis of Patient-Centredness Among Trauma and Orthopaedic Trainees in Wales

**DOI:** 10.7759/cureus.107971

**Published:** 2026-04-29

**Authors:** Deepika Pinto, Peter Cnudde, Stefan Bajada, Clare Carpenter

**Affiliations:** 1 Trauma and Orthopaedics, Cardiff and Vale University Health Board, Cardiff, GBR; 2 Trauma and Orthopaedics, Hywel Dda University Health Board, Camarthen, GBR; 3 Trauma and Orthopaedics, Hywel Dda University Health Board, Carmarthen, GBR

**Keywords:** medical education, orthopaedic curriculum, orthopaedic education, patient-centred care, person-centred care

## Abstract

Introduction: Patient-centred care (PCC) is a model of healthcare which encourages collaboration and shared decision-making (SDM) between patients, families and healthcare providers; to provide holistic and bespoke care. This study assessed the current state of PCC and its training among trainees on a Trauma and Orthopaedic (T&O) rotation in Wales, UK.

Methods: A survey was designed to elicit this in three domains: attitudes and self-perceived competencies; formal training and support; administrative support and barriers faced. After trialling in a pilot group, the questionnaire was electronically circulated among trainees and anonymised responses collected.

Results: 69% trainees (43/62) responded to the survey. Most believed that PCC was very important in orthopaedic surgery and had moderate-to-high self-perceived confidence in their abilities (abilities to communicate, address patient fears/anxieties, and provide culturally-sensitive care). Most respondents (>80%) ‘often’ or ‘always’ practiced SDM, elicited patient ideas/concerns/expectations, and considered lifestyles/goals when discussing treatments. There was moderate agreement among respondents that the current training programme adequately prepared them to provide PCC, although 74% reported they had not received any formal PCC training. Trainees expressed moderate agreement on receiving “support/guidance” and “constructive feedback on communication skills” from supervisors. 63% trainees expressed interest in training on PCC skills. “Adequate implementation of PCC practices in hospital policy/administration” generated mixed responses. High patient volumes, time constraints, lack of resources and systemic barriers were the commonly cited obstacles to providing PCC.

Conclusion: Orthopaedic trainees in Wales are cognisant of PCC and are reasonably confident in providing it, but would welcome further training and development of resources.

## Introduction

Patient-centred care (PCC) is a well-known concept. The American Academy of Orthopaedic Surgeons has defined it as “the provision of safe, effective, and timely musculoskeletal care achieved through co-operation between the orthopaedic surgeon, an informed and respected patient (and family), and a coordinated health care team” [1]. It includes strategies like shared decision-making (SDM), personalised care planning and self-management support [2].

PCC was the logical evolution from the earlier more traditional models of doctor-centred and disease-centred care. In the UK, the origin of PCC can be traced to the work of Michael and Enid Balint, who advocated for the physician to understand the patient as a whole person [3]. In the 1980s the Picker Institute - an organisation dedicated to the promotion of a patient-centred approach in healthcare - collaborated with researchers at Harvard Medical School, and through the use of extensive focus groups including recently discharged patients, family members and healthcare staff, developed the eight Picker principles of PCC [4,5]. In 2001, a landmark report by the Institute of Medicine included PCC among its six aims for improving the quality of healthcare, thus bringing wider recognition to this concept [6].

Another similar concept is that of ‘person-centred care’, which puts the person at the centre, taking into consideration their background, family and individual strengths and weaknesses [7]. While the terms 'PCC' and ‘person-centred care’ are often used interchangeably, they represent distinct conceptual frameworks. PCC traditionally focuses on the clinical encounter and functional outcomes, whereas person-centred care emphasises long-term relationships and holistic life meaning [7,8]. This study adopts the construct of PCC as its primary framework and utilises this terminology throughout. This choice aligns with the episodic nature of orthopaedic training and the specific focus of this study on the trainee-patient interaction. While we acknowledge the conceptual overlap and the use of the term 'person-centred care' in some of the cited literature, this study primarily deals with the shift away from traditional paternalistic (doctor- or disease-centred) models-a shift fundamentally captured by the PCC framework.

PCC is now a key theme in health policies around the world. In the National Health Service (NHS) within Wales, patient-centredness is one of the 12 Health and Care Quality Standards [9]. While the principles of PCC may seem like common sense in the provision of healthcare, it is not necessarily standard practice [10]. There is still the need to break away from the traditional approaches and shift the focus from “what’s the matter” with patients to “what matters” to them [10].

In recent years, there has been an increasing recognition of the importance of PCC within the specialty of orthopaedics [1,10]. Studies have demonstrated better outcomes and greater patient satisfaction when PCC principles are followed as compared to previous more traditional models of healthcare [11-13]. For example, Olsson et al demonstrated significantly reduced length of hospital stay following total hip arthroplasty among patients managed with a PCC approach versus a control group treated according to standard clinical practice [12]. Furthermore, it is evidenced that training in PCC skills could potentially improve the delivery of PCC by healthcare professionals [14].

Given its importance, PCC should be actively taught in undergraduate medical and postgraduate specialty courses. Educational bodies such as the Royal College of Surgeons, which determines the scope and curriculum of orthopaedic training in the UK, have incorporated PCC concepts in their specialty curricula, however it has remained unclear how this is implemented and assessed by individual deaneries [2].

The aim of this study was to assess the current perceptions of PCC among the trainees on a Trauma and Orthopaedic (T&O) rotation in the NHS Wales, UK. This was assessed in three domains: trainee attitudes towards PCC and their self-perceived competencies in providing it; the formal training and support from supervisors that trainees received for providing PCC; and finally, the administrative support for PCC and the barriers trainees faced in the provision of PCC.

## Materials and methods

A survey questionnaire was designed to explore trainees’ attitudes and experiences in providing PCC, and any relevant training they had received. The survey had three sections. The first collected anonymised demographic information, including the orthopaedic sub-specialties the trainees had been exposed to and expressed interest in. The second section focused on their attitudes towards PCC: whether they valued it and considered it an important part of providing T&O care, self-assessment of their capabilities in providing PCC and the perceived barriers to the provision of PCC. The last section focused on an assessment of the training programme: whether trainees felt adequately supported in providing PCC, the PCC-related training they received or felt they needed, and the guidance and feedback they received on the provision of PCC. 

The questionnaire was designed to touch upon various aspects of PCC, using the Picker principles as a broad guide. Table 1 enumerates the eight Picker Principles of PCC. The questions were a mixture of multiple-choice questions and Likert-scale-based assessments. Table 2 shows how responses from the 10-point Likert scales were interpreted for the purposes of this study. Additionally, some questions were open-ended, providing the opportunity to raise points not already covered by the survey. All survey items (apart from the open-ended questions) were mandatory, thus avoiding incomplete or selective responses. A copy of the survey questionnaire is provided in Appendix 1.

**Table 1:** The Picker principles of PCC [4] PCC: Patient-centred care

The Picker principles of PCC	
1	Clear information, communication and support for self-care
2	Effective treatment by trusted professionals
3	Emotional support, empathy and respect
4	Involvement in decisions and respect for preferences
5	Involvement and support for family and carers
6	Attention to physical and environmental needs
7	Fast access to reliable healthcare advice
8	Continuity of care and smooth transitions

**Table 2:** Interpretation of 10-point Likert scale questions

Score	Interpretation
1-3	Very low agreement
4-6	Low agreement
7-8	Moderate agreement
9-10	High agreement

The main target for the survey was the T&O registrars - both specialty trainees (STs) and specialist, associate specialist and specialty doctors (SAS doctors). The survey was first administered to a pilot group of five doctors - a mixture of core surgical trainees and senior house officers - with declared interests in pursuing T&O specialty training. The purpose of this was to analyse the questionnaire for clarity and ease of answering the questions, and to check for any discrepancies or ambiguities. This initial input was only used to fine-tune the survey, and responses from the pilot group were not included in the later analysis. The final version, after approval from senior authors, was administered to the target cohort of T&O Registrars. 

All responses were collected anonymously. The survey was administered using the Google Forms platform, between 12 February and 13 March 2025, thus allowing adequate time for response. A single reminder was sent out halfway through this period to encourage participation. Responses were restricted to one per participant.

Data analysis was performed using Microsoft Excel software and descriptive statistics have been used to summarise the findings. As all survey items were mandatory, the denominator for all calculations remained constant. Categorical variables are presented as percentages, while ordinal survey responses (e.g., Likert scales) are reported as medians with interquartile ranges (IQRs). Qualitative data from open-ended questions were analysed using a thematic approach, in which responses were categorised into emergent themes and reported narratively to supplement the quantitative findings.

The study was conducted in accordance with the UK Health Research Authority (HRA) framework for health-related research. Following an assessment via the HRA Decision Tool, the project was classified as a service evaluation of medical education. In line with local institutional governance procedures, it was determined that formal Research Ethics Committee (REC) review was not required. This was based on the study's design as an anonymous survey of healthcare staff that did not influence patient care or the training progression of the participants.

Participants were contacted via institutional work emails. The survey landing page provided information about the study and implied informed consent was obtained through the voluntary completion and submission of the survey. To ensure participant anonymity, no identifiable personal data were recorded.

## Results

**Figure 1:**
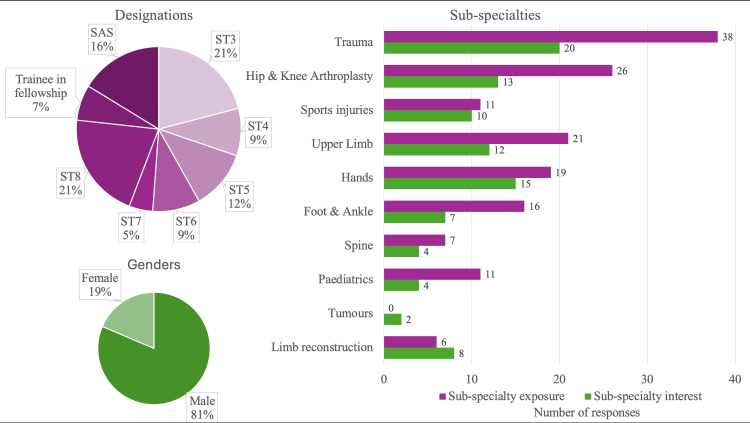
Trainee demographics - designations, genders and sub-specialty exposure/interests The data labels in the bar chart signify the number of responses by trainees for each sub-specialty. For each sub-specialty, the purple bar indicates the number of trainees that responded that they had exposure to that sub-specialty, the green bar indicates the number of trainees that responded that they had an interest in that sub-specialty for future career progression. The number following “ST” signifies the year of training in T&O. ST: Specialty trainee; SAS: Specialist, associate specialist and specialty doctors; T&O: Trauma and Orthopaedics

Of the 62 trainees invited to participate in the survey, 43 (69%) responded to the questionnaire. This included 35 males and eight females, with a wide distribution of training levels (Figure 1). Most trainees had significant exposure to mainly trauma and hip and knee arthroplasty in their training thus far, with relatively lesser exposure to other sub-specialties. Trauma, hands and arthroplasty were the most common sub-specialties of interest for future career progression. The demographic characteristics of the trainees surveyed are summarised in Figure 1.

Trainee attitudes and self-perceived competencies

**Figure 2:**
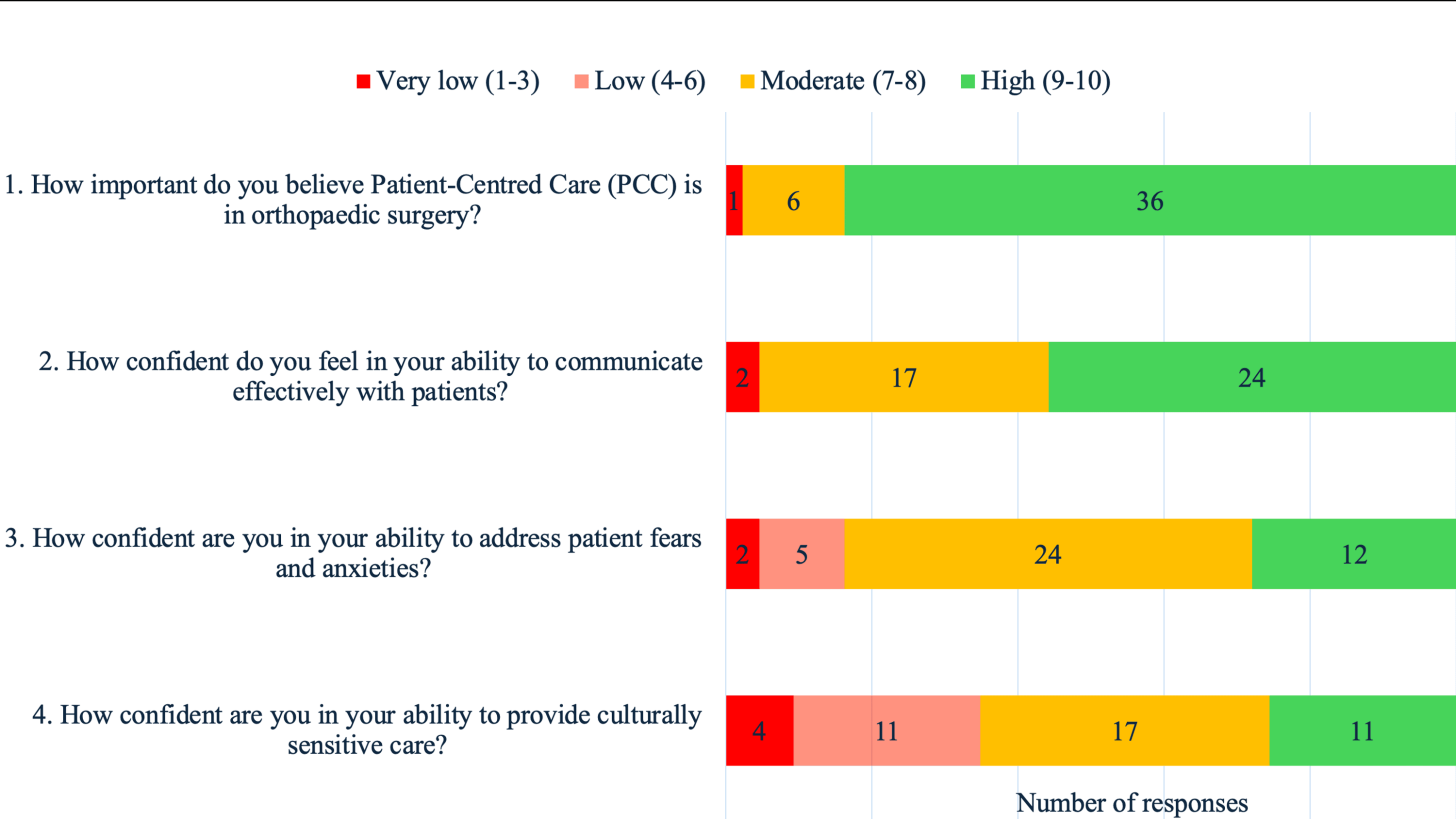
Trainee perspectives on PCC and their self-perceived competencies in providing it For each question, respondents selected a response on a scale of one to 10, one indicating the lowest level of agreement, and 10 indicating the highest. Based on this, scores 1-3 were interpreted as “very low”, 4-6 as “low”, 7-8 as “moderate” and 9-10 as “high”. The data labels indicate the number of responses belonging to each response category (i.e.“very low”, “low”, “moderate” or “high”). PCC: Patient-centred care

**Figure 3:**
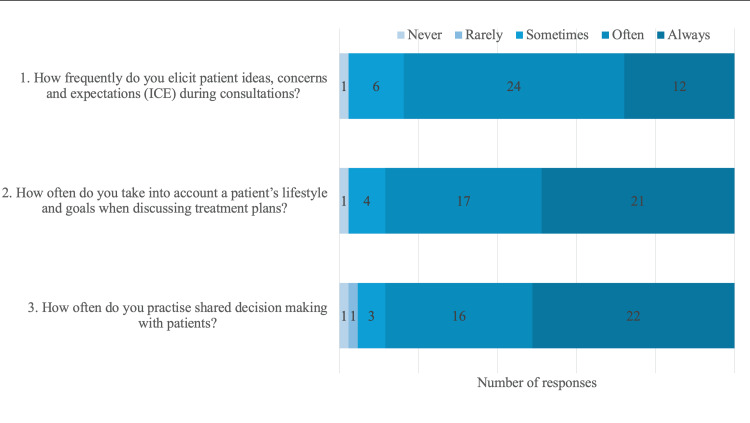
Further trainee self-assessments of their PCC practice For each question, respondents selected a response on a five-point scale consisting of “never”, “rarely”, “sometimes”, “often” and “always”. The data labels indicate the number of responses for each response category (i.e., “never”, “rarely”, “sometimes”, “often” or “always”). PCC: Patient-centred care

Most of the surveyed trainees (84%) believed that PCC is very important in orthopaedic surgery (Figure 2). Perceived confidence levels in delivering PCC were assessed using Likert scale-based questions, with responses ranging from one to 10, with higher scores indicating higher levels of confidence. According to this scale, median scores of nine, eight and seven, respectively, were reported for trainees’ self-perceived abilities to “communicate”, “address patient fears/anxieties”, and “provide culturally-sensitive care” (IQR 8-10, 7.5-9 and 6-8.5, respectively), thus suggesting moderate to high levels of confidence in these abilities (Figure 2). Most trainees reported that they “always” (28%) or “often” (56%) elicited patients’ ideas, concerns and expectations (ICE) during consultations, and the majority “always” (49%) or “often” (40%) took into account patients’ lifestyles and goals when discussing treatment plans (Figure 3). Similarly, most trainees “always” (51%) or “often” (37%) practised SDM with patients. It is important to point out that this data reflects the trainees’ perceptions of their PCC practice and not verified PCC performance.

Formal training and support from supervisors

**Figure 4:**
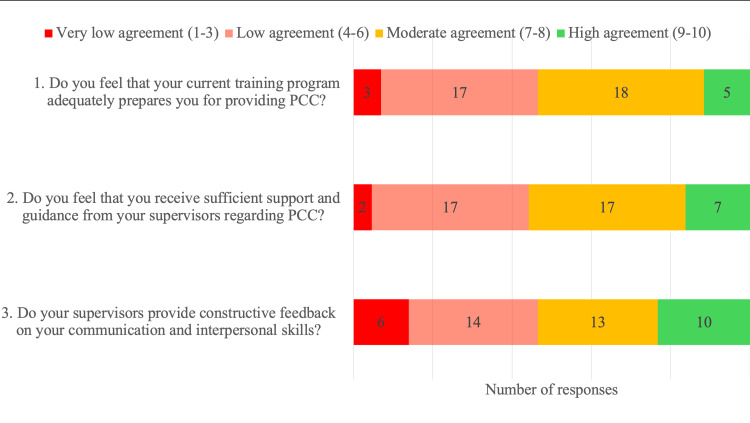
Training support for PCC For each question, respondents selected a response on a scale of one to 10, one indicating the lowest level of agreement, and 10 indicating the highest. Based on this, scores 1-3 were interpreted as “very low agreement”, 4-6 as “low agreement”, 7-8 as “moderate agreement” and 9-10 as “high agreement”. The data labels indicate the number of responses belonging to each response category (i.e.“very low agreement”, “low agreement”, “moderate agreement” or “high agreement”). PCC: Patient-centred care

Notably, 74% of trainees responded that they had not received any formal training on PCC, and those that had received training in PCC stated that it was during their undergraduate training. Respondents felt that “some” (54%) to “all/most” (30%) of their supervisors practiced PCC. We used a further set of Likert scale-based questions to assess the support trainees received from their supervisors for providing PCC. The responses ranged from one to 10, with one signifying the lowest level of agreement and ten signifying strong agreement. Regarding “support and guidance provided by supervisors”, and “receiving constructive feedback on communication and interpersonal skills”, the median scores were seven for both (IQR 5-8 for both), indicating low to moderate agreement (Figure 4). When asked “whether their current training programme adequately prepared them to provide PCC”, the median response was seven (IQR 5-8), again indicating low to moderate agreement.

When asked about interest in receiving training on PCC skills, 27 trainees (63%) responded that they would be interested in this, with eight (18.6%) responding in the negative and eight (18.6%) being unsure. Courses/workshops (47%), simulation exercises (37%), webinars (33%), mentorship programmes (30%), and lectures (21%) were the preferred modalities for receiving training in PCC.

Organisational support and barriers faced

**Figure 5:**
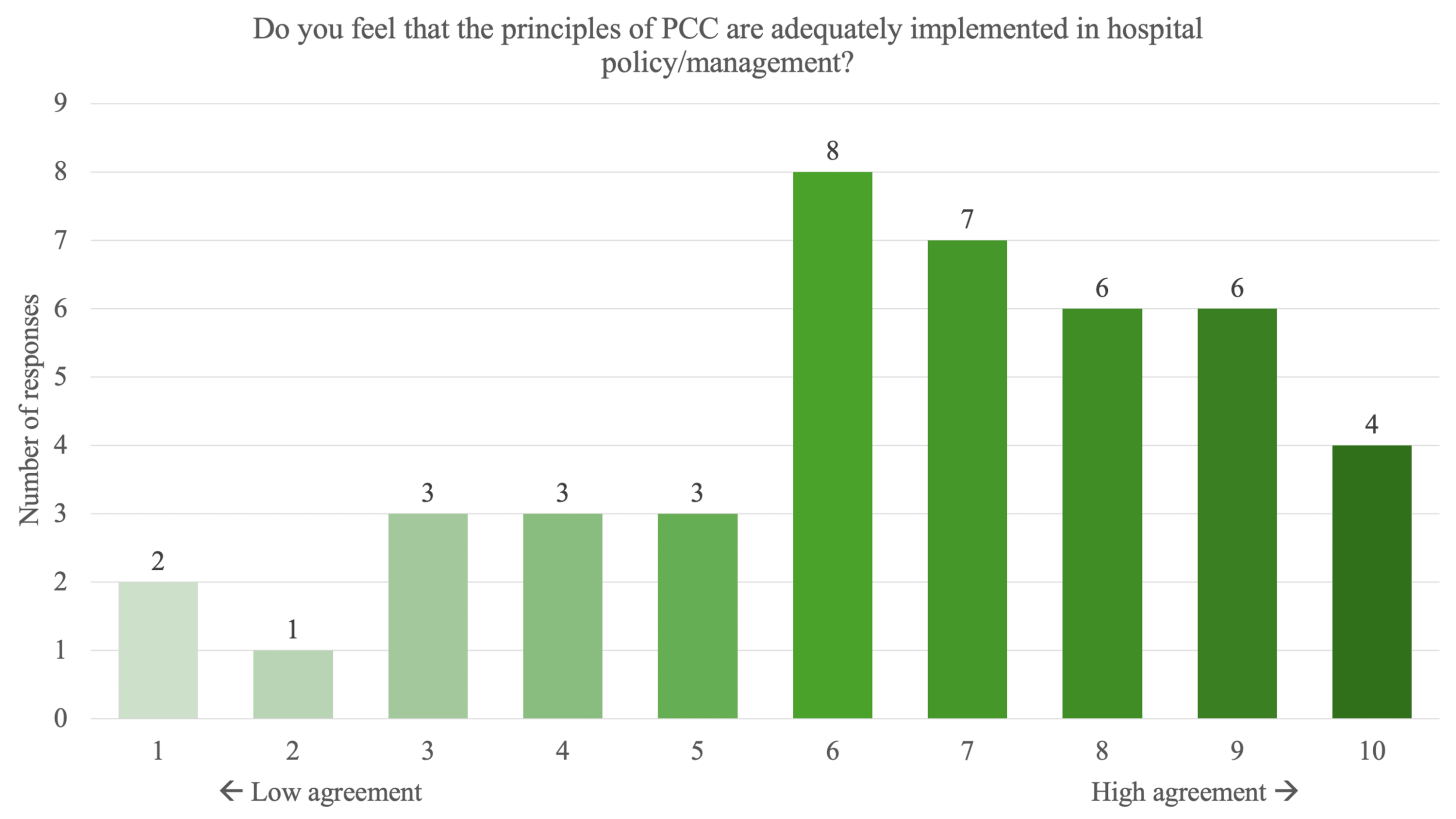
PCC implementation by hospital policy/management Trainees responded on a scale of one to 10, one indicating the lowest level of agreement and 10 indicating the highest, “Do you feel that the principles of PCC are adequately implemented in hospital policy/management?”. Scores 1-3 were interpreted as “very low agreement”, 4-6 as “low agreement”, 7-8 as “moderate agreement” and 9-10 as “high agreement”. The data labels indicate the number of responses for each value on the scale. PCC: Patient-centred care

**Figure 6:**
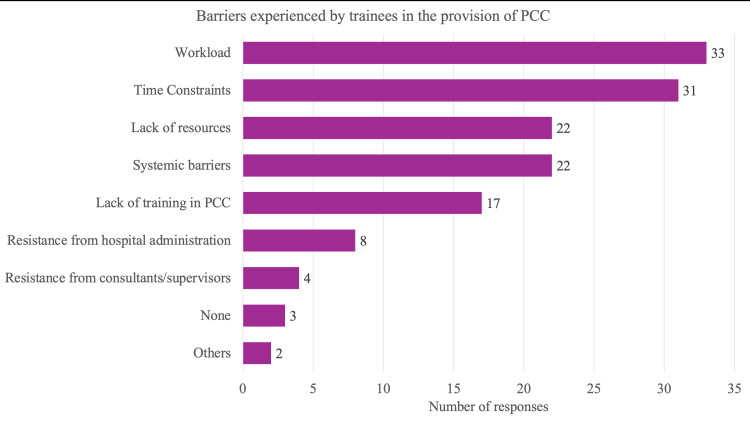
Barriers to providing PCC Trainees were asked the choose one or more from a list in response to the question, “What are the barriers you have experienced to providing PCC?”. The data labels indicate the number of responses from trainees for each option. “Lack of resources”: E.g., interpreters, patient information materials, etc. “Systemic barriers”: E.g., lack of clear guidelines, poor communication between departments, etc. “Workload”: High patient volumes “Others”: Free text responses - “lack of cultural competency training” and “paperwork and documentation requirements that can affect time spent with patients”. PCC: Patient-centred care

When asked whether PCC practices were adequately implemented in hospital policy/administration, responses were considerably spread-out with 14% being in very low agreement, 33% in low agreement, 30% in moderate agreement, and 23% in high agreement (Figure 5). When asked about the barriers faced when providing PCC, high patient volumes (77%), time constraints (72%), lack of resources like interpreters and patient information materials (51%) and systemic barriers like lack of clear guidelines and poor inter-departmental communication (51%) were the most commonly cited problems (Figure 6). Other issues included lack of training in PCC (40%), resistance from hospital administration (19%) and resistance from supervisors (9%). 

Other findings

Few additional points were raised by respondents when asked open-ended questions. Three respondents used this space to further emphasise that limited consultation times and overbooked clinics were obstacles to the provision of some aspects of PCC. Another noted that paperwork and documentation requirements can affect time spent with patients thus negatively influencing PCC. There was also a suggestion that “cultural competency training (including healthcare/body views) for common non-dominant ethnicities in the UK” would be helpful. Additionally, it was noted that attending clinics with supervisors and teaching sessions in hospitals could be counted as PCC training. Finally, there were further calls for better training in PCC, including assessment of skills in real-time scenarios as a part of trainees’ portfolios.

## Discussion

PCC is undeniably enshrined in health policy in the UK and around the world. However, there has been very little work exploring trainee doctors’ perceptions and how they acquire PCC skills [15]. This study provides an insight into PCC practices among T&O trainees in a typical UK rotation. Respondents in this study demonstrated generally favourable attitudes towards PCC, moderate-to-high self-perceived confidence in their ability to provide it, and a perceived unmet need for more structured training in PCC.

To the best of our knowledge, this appears to be one of the few studies exploring PCC perceptions among UK T&O trainees. Among our cohort, self-perceived confidence levels in communication and rapport-building skills were high. Whilst this was reassuring, it is important to acknowledge that this study has only analysed the delivery of PCC from the trainees’ perspectives. Studies show that there is often a discrepancy between healthcare providers’ and patients’ perceptions of PCC, with patients rating it lower than clinicians, particularly in domains like adhering to patient preferences and involvement of patients in their care [16]. Further studies surveying patients themselves are needed to get a true understanding of PCC delivery in this orthopaedic service. It must also be emphasised that our results rely on self-reported behaviours and self-rated competencies and are thus highly vulnerable to social desirability bias.

Most respondents confirmed that they had not received formal training on PCC during their T&O training programme. However, it must be recognised that while it may not be specifically labelled as “PCC training”, the underlying principles of treating patients with dignity, compassion and respect, and providing care that is personalised, coordinated and enabling, are at the core of T&O services in NHS Wales, and are integrated into the specialty training programme. The various aspects of PCC are included in the Generic Professional Capabilities (GPC) Framework, which enumerates the essential capabilities underlying professional medical practice in the UK [17]. The GPCs are a part of the curriculum for T&O specialty training, and are assessed periodically, in equal weightage to the Capabilities in Practice (CiPs) that define the professional skills trainees must master [18]. Furthermore, the GPCs are in keeping with “Good Medical Practice”, which is the framework of professional standards expected of all those registered with the General Medical Council (GMC), the regulatory body for doctors in the UK [19]. The findings in this study were similar to a previous study of UK medical speciality trainees, where the majority of doctors reported receiving no formal training in PCC skills, but acknowledged that most skills were picked up “on the job” [15]. There was some variation in the responses of our trainees regarding the practice of PCC by their supervisors and the support they received in developing these skills. However, the majority were satisfied in this respect. Patel et al. similarly noted that some senior consultants persist with old paternalistic models of care [15], which may explain some of the variability reported in our study. The data suggests that training in PCC skills should be offered to orthopaedic surgeons at all levels in order to standardise practice.

Respondents expressed interest in receiving formal training in PCC skills. There are numerous reports in the literature of how training in various PCC skills, in particular communication skills, can improve physician-patient interactions and patients’ satisfaction with care [20-22]. Such training has also been shown to counteract the decline in empathy that is otherwise seen during specialty training [14,23]. Providing trainees the opportunity to practice the relevant skills in a controlled environment such as workshops and receive constructive criticism are important strategies that should be incorporated into training programmes [24]. The Non-Technical Skills for Surgeons (NOTSS) course is a good example of how soft skills like communication, decision-making and situational awareness can be both taught and assessed [25].

This study has highlighted several barriers to the provision of PCC. As per previous reports in literature, barriers to the provision of PCC can be categorised as organisation-, provider- and patient-related [26,27]. Organisation-related barriers include time constraints and excessive workloads, lack of resources like interpreters and decision aids, policies and guidelines contrary to PCC, poor inter-departmental communication and co-ordination, and lack of leadership support for PCC practices. Provider-related barriers include rigid attitudes and beliefs which favour the traditional physician-centred model of healthcare, lack of awareness and training in the requisite skills, and compassion fatigue and burnout. Patient-related barriers include low levels of health literacy and variation in preference for participating in PCC. Tackling organisational issues like inadequate staffing, time constraints and excess workload have been demonstrated to improve the delivery of PCC [28,29] and were some of the key issues raised in this study.

This study had some limitations. There may be a degree of self-selection bias, as we have only captured the opinions of those who chose to participate in the study. However, our response rate of 69% is higher than the average for surveys of health professionals [30] and has likely captured the majority opinion in our cohort. Secondly, this study has provided a snapshot of PCC-related attitudes among trainees at one point in time. However, these attitudes and trainees’ skills are likely to evolve as they progress through training. Surveys such as this one must therefore be repeated at different intervals during training, and particularly after the implementation of interventions to bolster PCC-related skills so as to confirm their effectiveness. The inclusion of both STs and SAS doctors makes the surveyed group a heterogenous cohort, as their exposure to formal teaching, supervision and appraisal structures can differ significantly. Furthermore, we have sampled just one Deanery, and the results may not be generalisable to orthopaedic trainees in other parts of the UK, or indeed in other parts of the world. Even within the Welsh deanery, there is a wide geographical spread of individual hospitals, encompassing seven health boards and a wide socio-economic demographic. Some of the variation in responses from our trainees may be down to their variable institutional exposure during specialty training, or on where they received their undergraduate training. However, the circulation of the registrars to various hospitals during their training is a strength of the programme, exposing them to a variety of skills and attitudes, which they can eventually incorporate into their practice as consultants. Finally, we used a de novo questionnaire for this study, and though it did undergo pilot testing, it lacked formal psychometric validation (such as for content validity, face validity, reliability or internal consistency).

## Conclusions

Trainees on the Welsh T&O rotation are cognisant of PCC and perceive themselves as reasonably confident in providing it. Trainees at all levels report interest in further training in PCC and would welcome the development of resources for its provision. Barriers to the delivery of PCC, particularly organisational ones, must be overcome or circumvented. Further studies are needed to establish actual PCC competencies among trainees rather than perceived competencies, and to assess PCC practice and training in other deaneries and among other specialties. Patient input must also be sought to assess the quality of care provided and ensure that providers’ perceptions of care align with those of their patients.

## Appendices

Appendix 1

**Table 3:** Survey on PCC among T&O trainees in Wales The number following “ST” signifies the year of training in T&O. ST: Specialty trainee; PCC: Patient-centred care; T&O: Trauma and Orthopaedics; ICE: Ideas, concerns and expectations; SDM: Shared decision-making

	Question	Response options
1	What is your designation?	ST3, ST4, ST5, ST6, ST7, ST8, Trust Spr, Other (Select any one)
2	What is your gender?	Male, Female (Select any one)
3	Which orthopaedic sub-specialties have you had significant exposure to in your training so far?	Trauma, Hip and Knee Arthroplasty, Sports injuries, Upper Limb, Hands, Foot & Ankle, Spine, Paediatrics, Tumours, Limb reconstruction, Other (Select all that apply)
4	Which is/are your Orthopaedic sub-specialties of interest?	Trauma, Hip and Knee Arthroplasty, Sports injuries, Upper Limb, Hands Foot & Ankle, Spine, Paediatrics, Tumours, Limb reconstruction, Other (Select all that apply)
5	How important do you believe PCC is in orthopaedic surgery?	Select a number on a scale of 1 to 10, 1 indicating “not at all important” and 10 indicating “very important”.
6	How confident do you feel in your ability to communicate effectively with patients? (e.g., using jargon-free language, using diagrams/models to explain concepts etc.)	Select a number on a scale of 1 to 10, 1 indicating “very weak” and 10 indicating “very strong”.
7	How frequently do you elicit patient ICE during consultations? (We realise that this is not always possible in the context of trauma, so please focus on elective clinics for this question.)	Always, Often, Sometimes, Rarely, Never (Select any one)
8	How often do you take into account a patient’s lifestyle and goals when discussing treatment plans? (E.g., Vocational and recreational activities that may be affected by the disease process or its treatment?)	Always, Often, Sometimes, Rarely, Never (Select any one)
9	How often do you practise SDM with patients? (SDM is defined as a joint process in which a healthcare professional works together with the patient to reach a decision about care.)	Always, Often, Sometimes, Rarely, Never (Select any one)
10	How confident are you in your ability to address patient fears and anxieties?	Select a number on a scale of 1 to 10, 1 indicating “very weak” and 10 indicating “very strong”.
11	How confident are you in your ability to provide culturally sensitive care? (E.g., use of interpreters for non-English speaking patients, discussing use of grafts where there might be restrictions based on religion/culture etc.)	Select a number on a scale of 1 to 10, 1 indicating “very weak” and 10 indicating “very strong”.
12	Do you feel that PCC is routinely practiced by your supervisors?	All/most, Some, Few, Very few/none, Not sure (Select any one)
13	Do you feel that the principles of PCC are adequately implemented in hospital policy/management? (E.g., Personalised care plans, Patient initiated follow up, etc.)	Select a number on a scale of 1 to 10, 1 indicating “strongly disagree” and 10 indicating “strongly agree”.
14	What are the barriers you have experienced to providing PCC?	Time constraints, Workload (high patient volumes), Lack of resources (e.g., interpreters, patient information materials), Lack of training in PCC, Resistance from consultants/supervisors, Resistance from hospital administration, Systemic barriers (e.g., lack of clear guidelines, poor communication between departments), None, Other (Select all that apply)
15	Are there any aspects that you feel you need to work on to provide better PCC? (E.g., less focus on the computer, more focus on the patient etc.)?	Free text response
16	Have you received any formal training in PCC?	Yes, No, Not sure (Select any one)
17	If yes, please specify the type of training:	Free text response
18	Do you feel that your current training program adequately prepares you for providing PCC?	Select a number on a scale of 1 to 10, 1 indicating “strongly disagree” and 10 indicating “strongly agree”.
19	What type of training do you feel would be most beneficial to you to improve your PCC skills?	Courses/workshops, Lectures, Simulation exercises, Webinars, Mentorship programmes, None required, Other (Select all that apply)
20	Do you feel that you receive sufficient support and guidance from your supervisors regarding PCC?	Select a number on a scale of 1 to 10, 1 indicating “strongly disagree” and 10 indicating “strongly agree”.
21	Do your supervisors provide constructive feedback on your communication and interpersonal skills?	Select a number on a scale of 1 to 10, 1 indicating “strongly disagree” and 10 indicating “strongly agree”.
22	Would you be interested in further training on PCC, such as workshops or online modules?	Yes, No, Not sure (Select any one)
23	Are there any other ways in which you can be supported to deliver PCC?	Free text response
